# Substrate Shift Reveals Roles for Members of Bacterial Consortia in Degradation of Plant Cell Wall Polymers

**DOI:** 10.3389/fmicb.2018.00364

**Published:** 2018-03-01

**Authors:** Camila Carlos, Huan Fan, Cameron R. Currie

**Affiliations:** ^1^Department of Bacteriology, University of Wisconsin–Madison, Madison, WI, United States; ^2^U.S. Department of Energy, Great Lakes Bioenergy Research Center, University of Wisconsin–Madison, Madison, WI, United States

**Keywords:** microbial enrichment, lignocellulose, xylan, lignin, *Pseudomonas*, *Sphingobacterium*

## Abstract

Deconstructing the intricate matrix of cellulose, hemicellulose, and lignin poses a major challenge in biofuel production. In diverse environments in nature, some microbial communities, are able to overcome plant biomass recalcitrance. Identifying key degraders of each component of plant cell wall can help improve biological degradation of plant feedstock. Here, we sequenced the metagenome of lignocellulose-adapted microbial consortia sub-cultured on xylan and alkali lignin media. We observed a drastic shift on community composition after sub-culturing, independently of the original consortia. Proteobacteria relative abundance increased after growth in alkali lignin medium, while Bacteroidetes abundance increased after growth in xylan medium. At the genus level, *Pseudomonas* was more abundant in the communities growing on alkali lignin, *Sphingobacterium* in the communities growing on xylan and *Cellulomonas* abundance was the highest in the original microbial consortia. We also observed functional convergence of microbial communities after incubation in alkali lignin, due to an enrichment of genes involved in benzoate degradation and catechol ortho-cleavage pathways. Our results represent an important step toward the elucidation of key members of microbial communities on lignocellulose degradation and may aide the design of novel lignocellulolytic microbial consortia that are able to efficiently degrade plant cell wall polymers.

## Introduction

Plant biomass is renewable and rich in energy, and as such, is a promising fuel alternative to fossil fuels (Goldemberg, 2009; Liao et al., 2016). However, low efficient plant cell wall deconstruction is a major barrier for economically viable biofuel production (Klein-Marcuschamer and Blanch, 2015). Plant cell walls are composed of a matrix of heteropolymers (i.e., cellulose, hemicellulose, and lignin) that are physically structured to prevent enzymatic breakdown. Commonly, a chemical and/or physical pretreatment of lignocellulosic material is applied to make cellulose accessible to hydrolytic enzymes through the solubilization of hemicelluloses and/or lignin (Mosier et al., 2005).

Natural or engineered microbial consortia are a promising approach to overcoming plant biomass recalcitrance, because of their ability to extract the majority of the energy from plant polymers in nature (Wei et al., 2009; Gessner et al., 2010; Lewin et al., 2016). The enzymatic degradation of plant material depends on multiple biomass-degrading enzymes, with complementary and synergistic activities, which are secreted by various species of micro-organisms (Allgaier et al., 2010; DeAngelis et al., 2010; Cragg et al., 2015; Lemos et al., 2017). Microbial enrichment in specific lignocellulose material is a common approach to obtain efficient and simple microbial assemblages that are able to degrade plant cell wall components (de Lima Brossi et al., 2016; Jiménez et al., 2016; Korenblum et al., 2016; Maruthamuthu et al., 2016; Mello et al., 2016). Cellulose-degrading consortia have been obtained from leaf-cutter ant refuse dumps (Lewin et al., 2016), and mixtures of plant material and chicken feces (Haruta et al., 2002). Lignin-degrading consortia have been obtained from wetland (Wang et al., 2013), rice paddy field soil (Kato et al., 2015), and redwood compost (Ceballos et al., 2017). When compared to the environmental inoculum, the enrichment derived consortia show reduced diversity, as a result of the limited number of ecological niches and selection toward more efficient degraders (Jiménez et al., 2017). The taxonomic and functional composition of lignocellulolytic microbial consortia are mainly influenced by the community in the environmental inoculum, and the substrate used for the enrichment (Cortes-Tolalpa et al., 2016; de Lima Brossi et al., 2016; Wong et al., 2016). For example, Wong et al. (2016) compared microbial enrichments derived from beaver dropping and moose rumen and found that the substrate is a major driver of the microbial consortia composition. Jiménez et al. (2016) used forest soil as an inoculum and also found distinct taxonomic composition in bacterial communities enriched in wheat straw, switchgrass and corn stover, but with similar profiles of carbohydrate-active enzyme families. Interestingly, in these studies, even after several batch cultures, the resulting microbial consortia still presented dozens of bacterial species. In plant biomass degradation, coexistence of several microbial species is possible as different members of a consortium occupy different niches, i.e., have different carbon source preferences (Jiménez et al., 2017).

In this study, we aimed to identify members of microbial consortia that have specific preferences for two major components of plant cell wall, xylan and lignin. Eight plant biomass-degrading microbial consortia were subcultured in minimal medium with xylan or alkali lignin as the sole carbon source. Deep metagenomics sequencing of these communities revealed changes in the abundance of distinct bacterial members in response to xylan and lignin-derived compounds. Furthermore, we identified metabolic pathways that were enriched after the substrate shifting.

## Materials and Methods

### Enrichment Cultures

Three chicken feces and five soil samples were aseptically collected in DeForest, WI (N 43.204365, W -89.283446) in September 28, 2015 and stored at 4°C for 1 day until inoculation (**Table 1**). Samples were collected around 5 m from each other. Each sample was homogenized with sterile phosphate-buffered saline (PBS) and 10 μL of the solution was inoculated in 5 mL of autoclaved minimal medium with 0.025 g of poplar wood chips (size < 1 mm) or 1 cm × 10 cm strip of Whatman #1 filter paper. M63 minimal medium was used, containing 61.5 mM potassium phosphate, 38.5 mM potassium phosphate monobasic, 15.1 mM ammonium sulfate, 0.5 mg/L of iron sulfate, 1 mM magnesium sulfate solution, 1 mg/L of thiamine, and 5 mL/L of SPV-4 trace elements solution (Voelskow, 1988). Consortia were grown at 30°C at 250 rpm. After 7 days samples were vortexed, and 50 μL from each was transferred into tubes containing 5 mL of sterile M63 medium with one enrichment substrate (filter paper or wood chips). After 24 passages, these consortia were centrifuged at 12,000 *g* for 5 min and the pellets were stored at -80°C until DNA extraction. Two microliter of these consortia were inoculated to 3 mL of autoclaved M63 medium containing 5% (w/v) of beechwood xylan (Megazyme) or alkali lignin with low sulfonate content (Sigma-Aldrich) and incubated at 30°C at 250 rpm for 7 days. Cultures were centrifuged at 12,000 *g* for 5 min and the pellets were maintained at -80°C until DNA extraction.

**Table 1:** Description of the metagenomic samples used in this study.

Sample name	Treatment	Inoculum	Enrichment substrate	Total number of reads passed QC	Total number of bases in contigs	Total gene count	IMG ID	IMG Name
S1	Original	Soil	Filter paper	14,071,782	70,033,963	95,832	3300012821	DID2877_E24
S1.Lig	Lignin	Soil	Filter paper	13,863,076	61,021,420	80,653	3300012804	DID2877_E24_Lignin
S1.Xyl	Xylan	Soil	Filter paper	13,206,130	66,984,622	80,990	3300012811	DID2877_E24_Xylan
S2	Original	Soil	Wood chips	15,279,458	78,306,800	112,814	3300012799	DID2878_E24
S2.Lig	Lignin	Soil	Wood chips	16,388,664	80,882,717	107,226	3300012801	DID2878_E24_Lignin
S2.Xyl	Xylan	Soil	Wood chips	17,105,142	54,474,056	77,518	3300012796	DID2878_E24_Xylan
C3	Original	Chicken feces	Wood chips	14,985,520	152,610,958	224,072	3300012862	DID2937_E24
C3.Lig	Lignin	Chicken feces	Wood chips	15,514,838	102,584,925	137,408	3300012840	DID2937_E24_Lignin
C3.Xyl	Xylan	Chicken feces	Wood chips	16,445,174	226,810,859	337,114	3300012879	DID2937_E24_Xylan
C1	Original	Chicken feces	Filter paper	14,249,310	87,284,368	112,117	3300012810	DID2882_E24
C1.Lig	Lignin	Chicken feces	Filter paper	13,511,478	63,445,060	87,862	3300012800	DID2882_E24_Lignin
C1.Xyl	Xylan	Chicken feces	Filter paper	12,633,900	72,633,020	96,433	3300012802	DID2882_E24_Xylan
C2	Original	Chicken feces	Filter paper	16,753,258	47,006,209	57,079	3300012823	DID2898_E24
C2.Lig	Lignin	Chicken feces	Filter paper	15,495,898	57,746,371	80,856	3300012817	DID2898_E24_Lignin
C2.Xyl	Xylan	Chicken feces	Filter paper	16,400,218	48,127,918	70,168	3300012797	DID2898_E24_Xylan
S3	Original	Soil	Wood chips	16,445,174	7,592,135	9,196	3300012789	DID2925_E24
S3.Lig	Lignin	Soil	Wood chips	7,817,840	10,282,172	15,310	3300012792	DID2925_E24_Lignin
S3.Xyl	Xylan	Soil	Wood chips	8,087,312	10,926,255	12,253	3300012791	DID2925_E24_Xylan
S4	Original	Soil	Wood chips	16,388,570	108,195,268	141,668	3300012842	DID2933_E24
S4.Lig	Lignin	Soil	Wood chips	23,490,534	92,910,293	128,456	3300012833	DID2933_E24_Lignin
S4.Xyl	Xylan	Soil	Wood chips	13,402,340	70,611,347	89,703	3300012808	DID2933_E24_Xylan
S5	Original	Soil	Wood chips	13,830,936	135,709,802	199,758	3300012859	DID2934_E24
S5.Lig	Lignin	Soil	Wood chips	9,743,042	67,969,669	112,711	3300012822	DID2934_E24_Lignin
S5.Xyl	Xylan	Soil	Wood chips	15,354,958	90,451,188	125,640	3300012830	DID2934_E24_Xylan

### Metagenome DNA Extraction Sequencing, Assembly, and Annotation

Metagenomic DNA was extracted using the PowerSoil^®^ DNA Isolation Kit (Mo Bio, Carlsbad, CA, United States) following the manufacturer instructions. The DNA was quantified using a Qubit Kit (Invitrogen, Carlsbad, CA, United States), and the integrity was confirmed by 1% agarose gel electrophoresis. DNA yields are shown in Supplementary Table S1. Metagenomic library construction and sequencing were carried out at the Joint Genome Institute^1^ using the Illumina HiSeq-2500 platform (2 × 150 bp). Read ends were trimmed to a minimum quality score of 12 and filtered to have a maximum of three ambiguous bases and a minimum length of 51. Trimmed and filtered paired-end reads were assembled using the Megahit assembler (Li D. et al., 2016) with the default setting except the following option:“–k-list 23,43,63,83,103,123.” Functional annotation and taxonomic classification were performed using the Integrated Microbial Genomes pipeline (Chen et al., 2016; Huntemann et al., 2016). The assembled metagenomes were deposited in the NCBI whole genome sequence (WGS) as BioProject PRJNA422409.

### Statistical and Bioinformatics Analyses

KEGG Orthologous (KO) distributions were normalized as counts per million reads (CPMR) calculated as (*R*/*N*)^∗^10^6^, where *R* is the number of mapped reads to a gene and *N* is the total number of reads of that sample. One-way analysis of similarity (ANOSIM) and principal coordinates analysis (PCoA), using Morisita index, were performed with PAST software version 3.15^2^. The significance of the relative abundance difference in KO was performed using Kruskal–Wallis and multiple test correction via Benjamini and Hochberg false discovery rate, which are implemented in the STAMP software version 2.1.3^3^. Relative abundance of bacterial genera in each sample was obtained by dividing the number of reads mapped to all the contigs classified to a genus by the total number of reads in each sample. ANOVA was used to test whether there are significant differences between the relative abundance of bacterial genera from those consortia. Contigs were assigned to the last common ancestor where at least 30% of the genes had USEARCH hits (Huntemann et al., 2016). Only contigs with 10 or more genes were used for this analysis. A network of association between bacterial genera and samples was visualized using Cytoscape v3.0 software.

### Cluster Analysis Based on *k*-mer Composition of Each Sample

In order to understand the relative similarities among the metagenomic datasets, we looked at the *k*-mer (short DNA strands with *k* base pairs) composition of each sample. We fed the contigs into a *k*-mer composition analysis pipeline, the AAF package (Fan et al., 2015). The distances used for clustering by the AAF package are the dissimilarities derived from the proportion of unique *k*-mers that are shared between each pair of samples. The parameters we used were *k* = 23 (the length of the *k*-mers) and *n* = 1 (minimum frequency of each *k*-mer to be considered present). *k* should be long enough to overcome the problem of homoplasy. We tested different *k* until the topology stabilized at 23. The minimum frequency is designed to filter out *k*-mers calculated from raw reads containing sequencing errors, which are usually less frequent than average sequencing depth. Because *k*-mers were calculated from assemblies in our case, *n* is set to 1, therefore no *k*-mer was filtered out.

We also looked at the Average Nucleotide Identity (ANI) between the assemblies of different metagenomic datasets, and used the dissimilarities (1-ANI) for the same type clustering analysis. ANI was calculated using pyani (Pritchard et al., 2016) with MUMmer (Kurtz et al., 2004) as the sequence aligner.

## Results

### Taxonomic Affiliation of Contigs and Single-Copy Genes

**FIGURE 1:**
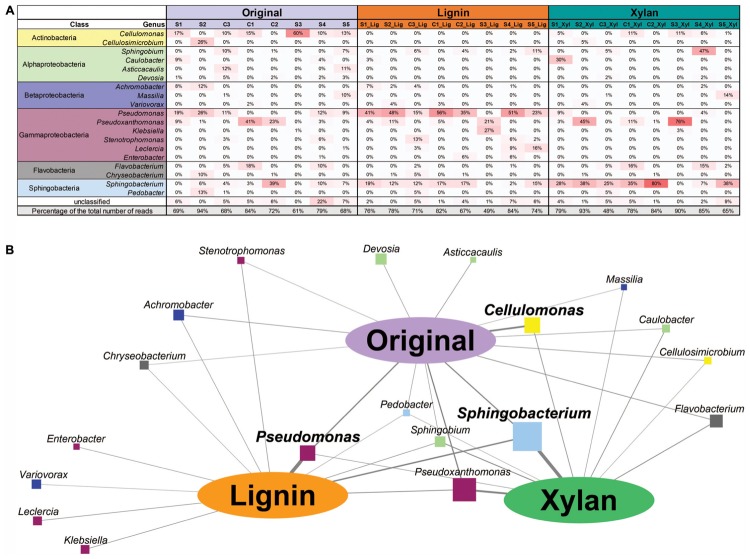
**(A)** Heatmap showing the relative abundance of the top bacterial genera assigned to contigs with 10 or more genes. **(B)** Association network between bacterial genera and carbon sources. Oval nodes represent carbon sources used in this study; squares nodes represent the top bacterial genera assigned to contigs with 10 or more genes; the size of the square node is proportional to the overall relative abundance of the bacterial genera across all the samples. Edge width represents the average relative abundance of the bacterial genera in each carbon source.

The 24 microbial consortia (eight original enrichments, eight subcultures in xylan, and eight subcultures in alkali lignin) were sequenced and a total of 52 Gb of high quality reads were obtained (**Table 1**). To identify the most abundant microorganisms growing on each carbon source, we used coverage information associated with the contigs classified by IMG to genus level (Markowitz et al., 2013). **Figure 1A** shows the relative abundance of each genus for the 20 most abundant genera found across the microbial consortia. In average, the relative abundance of *Pseudomonas* was higher after growth in alkali lignin medium than in the original consortia (ANOVA, *F* = 9.893, *p* = 0.007). Consortium S3_Lig showed an increase in the abundance of *Klebsiella* after growth in alkali lignin medium. *Cellulomonas* was not detected in any of the communities grown in alkali lignin medium and on average, it was not significantly different between xylan substrate and the original consortia. On average, *Sphingobacterium* abundance increased after growth in xylan medium when compared to the original consortia (ANOVA, *F* = 5.659, *p* = 0.032). *Pseudoxanthomonas* relative abundance increased after growth in xylan in consortium S3_Xyl, and *Sphingobium* increased in consortium S4_Xyl.

To further explore the association between bacterial genera and carbon source, a network was built, where the width of the edges represents the average abundance of each genus in each treatment (**Figure 1B**). The strongest associations were among *Sphingobacterium* and *Pseudoxanthomonas* with xylan, *Pseudomonas* with alkali lignin, and *Cellulomonas* with the original communities. Some genera were only associated with alkali lignin, such as *Klebsiella, Variovorax, Leclercia*, and *Enterobacter* and no genus was exclusively associated with xylan.

Community diversity was assessed via two single-copy genes (alanyl-tRNA synthetase: COG0013 and excinuclease ABC subunit B UvrB: COG0556) revealed similar taxonomic patterns (Supplementary Table S2). The diversity of COG0013 phylotypes varied from two to 19 per sample and the COG0556 varied from two to 17 phylotypes per sample. For both phylomarkers, no significant change in the number of phylotypes was found among the treatments.

### Microbial Community Shifts

**FIGURE 2:**
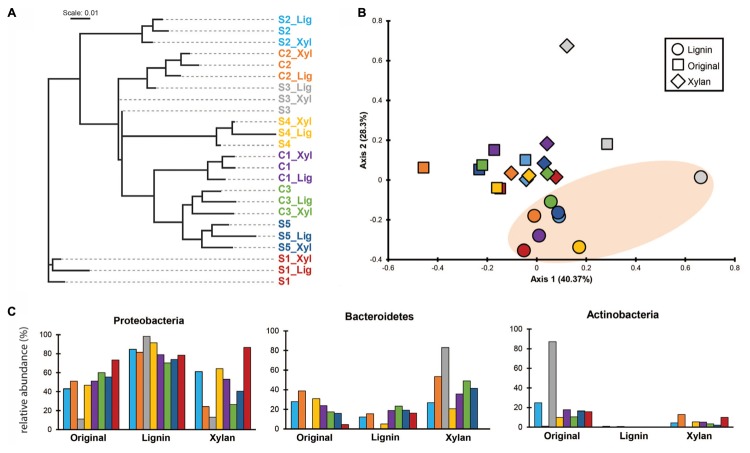
**(A)** Cluster analysis based on *k*-mer composition dissimilarities among samples (*k* = 23). Colors are used to distinguish enrichment communities. **(B)** PCoA based on Morisita’s overlap index calculated using counts per million reads of Kegg Orthologous on each sample. Oval orange shade was used to highlight the samples after growth in alkali lignin medium. **(C)** Relative abundance of genes assigned to major bacterial phyla in each sample. Taxonomic assignment was based on the top USEARCH hit.

Using *k-mer* composition, the microbial communities were clustered according to the enrichment community line (**Figure 2A**). The same trend is shown with the clustering based on ANI (Supplementary Figure S1). While their KO functional profiles revealed that the microbial communities are more similar to the ones with the same treatment (**Figure 2B**), with ones incubated in alkali lignin significantly more similar to each other than to their original communities, though communities after growth xylan medium did not changed significantly from the original communities (**Table 2**). Factors related to the history of the communities (inoculum, enrichment substrate and the enrichment community line) did not play a significant role on shaping the functional composition of the metagenomes (**Table 2**).

**Table 2:** One-way analysis of Morisita’s overlap index (ANOSIM) using counts per million reads of Kegg Orthologous.

	ANOSIM	
	*R*	*p*^∗^
**Factor**		
Treatment ^∗∗^	0.3213	0.0002
Enrichment substrate	–0.0432	0.6596
Enrichment community	0.05522	0.2245
Inoculum	–0.1016	0.9074
**Pairwise^∗∗^**		
Original, Lignin	0.3917	0.0006
Original, Xylan	0.1155	0.1059
Lignin, Xylan	0.4983	0.0003

*^∗^Bonferroni corrected. ^∗∗^Post hoc analysis. Treatment: Original, Lignin and Xylan. Enrichment substrate: Filter paper and wood. Enrichment community line: C1, C2, C3, S1, S2, S3, S4 and S5. Inoculum: Chicken feces and Soil.*

Taxonomic annotation of genes predicted from our metagenomic data to phylum level revealed that genes belonging to Proteobacteria dominated most of the original consortia, with exception of S3, which was dominated by genes identified as from Actinobacteria (87.1%) (**Figure 2C**). After growth in alkali lignin, all the consortia showed an increase in abundance of genes belonging to Proteobacteria, from an average of 48.9% to an average of 82.14%. When grown in xylan, on average the proportion of Bacteroidetes genes increased from 18.9 to 38.7%, with the exception of the consortia S2_Xyl, S1_Xyl and S4_Xyl (**Figure 2C**).

**FIGURE 3:**
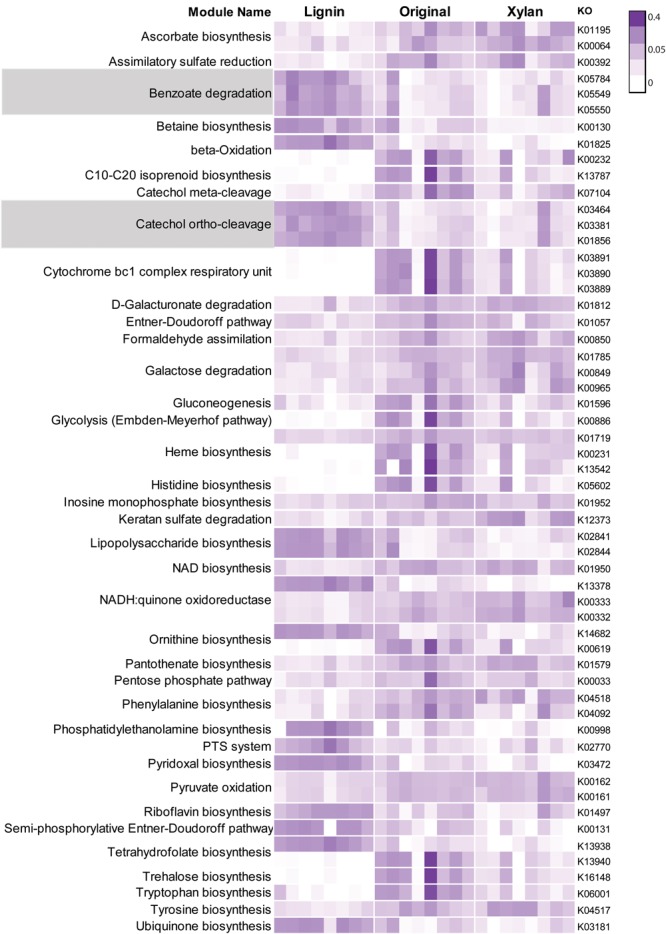
Heatmap showing Kegg modules with normalized counts significantly different (*p* < 0.05) among three treatments. Each row represent one Kegg Orthologous and each column represent one sample. Relative abundance values were normalized by the total sum of each row. Modules involved in the degradation of aromatic compounds are highlighted.

**FIGURE 4:**
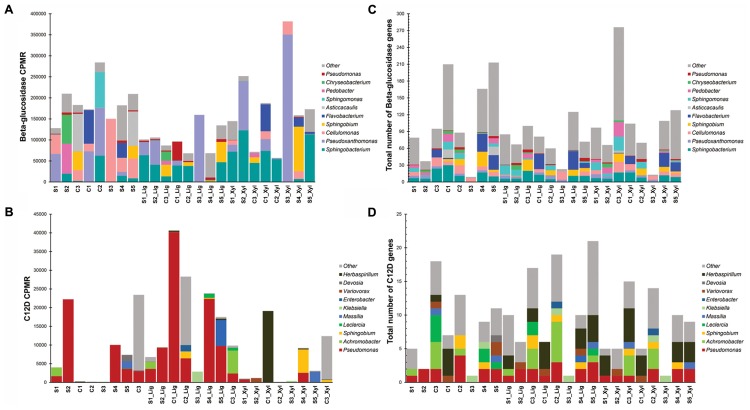
Distribution by genus of the number of genes **(A)** beta-glucosidase and **(B)** catechol 1,2-dioxygenase (C12D). Distribution of counts per million reads of genes **(C)** beta-glucosidase and **(D)** catechol 1,2-dioxygenase (C12D).

More than 270 functions were enriched after growth in alkali lignin (Kruskal–Wallis, corrected *p* < 0.05) and 399 functions were enriched in the original and/or xylan treatments (Kruskal–Wallis, corrected *p* < 0.05) (Supplementary Table S3). Functions involved in cellulose degradation were more abundant in the original consortia when compared to the alkali lignin consortia. These functions included cellulose 1,4-beta-cellobiosidases (K19668), endoglucanases (K01179), and beta-glucosidases (K05349 and K05350). Functions assigned to KEGG modules are shown in **Figure 3**. Genes involved in the catechol ortho-cleavage and benzoate degradation pathways were found to be overrepresented after growth in alkali lignin, while genes involved in pyruvate oxidation and galactose degradation were found overrepresented in the original and xylan samples. Catechol 1,2-dioxygenase (C12D) genes were predominantly classified to the genus *Pseudomonas* (**Figures 4C,D**). In contrast, beta-glucosidase genes were primarily classified to the genera *Sphingobacterium, Pseudoxanthomonas*, and *Cellulomonas* (**Figures 4A,B**).

## Discussion

Selective enrichment is a common approach for obtaining lignocellulose-degrading microbial consortia that can be used for biomass conversion to biofuels and other valuable biochemical. Studies have focused on identifying how inoculum sources (Cortes-Tolalpa et al., 2016) and enrichment conditions (de Lima Brossi et al., 2016; Wong et al., 2016) can affect the yield and efficiency of a lignocellulose-degrading microbial consortia. In our study, we aimed to identify community members responsible for the degradation of xylan and lignin-derived compounds. We expected that after incubation on xylan and alkali lignin, the resulting communities would be enriched in members that consume each of these substrates, independently of the history of the communities. We observed that after sub-culturing the consortia conserved the overall genetic repertoire of the original consortia, which was assessed by their *k-mer* composition, but the relative abundance of members carrying specific functions changed in response to the carbon source used, as observed with taxonomic and functional annotation.

The functional and taxonomic structure of the communities changed considerably after growth in alkali lignin, both in their taxonomic and functional profiles. Moreover, despite of their enrichment history, most of the consortia converged to a similar composition, with prevalence of *Pseudomonas* spp. and aromatic degradation genes. As mentioned in the introduction, substrate-driven convergence has already been observed in other studies (Jia et al., 2016; Wong et al., 2016). Jia et al. (2016) proposed that community convergence indicates that the selective pressure imposed by the culturing conditions is strong enough to change the initial relative abundance of community members. Lignin-derived compounds are known to be toxic to a various microorganisms, and may provide a competitive advantage to more tolerant species, such as *Pseudomonas* spp. (Abdelaziz et al., 2016). An increase in the abundance of *Pseudomonas* was associated with enrichment of genes related to aromatic compound degradation. Lignin is a heterogeneous aromatic polymer, which is found in the plant cell wall covalently cross-linked with polysaccharides (Li M. et al., 2016). Members of the *Pseudomonas* genus are considered major degraders of aromatic compounds in various environments (Ma et al., 2013; Terrón-González et al., 2016) and several studies have demonstrated that the *Pseudomonas* has the capability to catabolize low molecular weight lignin (Salvachúa et al., 2015; Tian et al., 2016; Mohan and Phale, 2017; Ravi et al., 2017). The strain *P. putida* CSV86 was shown to utilize aromatics, such as veratryl alcohol and ferulic acid, preferentially over glucose (Basu et al., 2006) via the ortho-cleavage pathway (Mohan and Phale, 2017). In our study, Catechol 1,2-dioxygenase (C12D) genes were enriched after growth with alkali lignin as a sole carbon source and were mostly attributed to *Pseudomonas* (**Figure 2**). C12D is the key enzyme in the ortho-cleavage pathway and catalyzes the intradiol cleavage of the aromatic ring generating *cis,cis*-muconic acid (Fuchs et al., 2011) and it is likely involved in the degradation of kraft lignin by *Cupriavidus basilensis* B-8 (Shi et al., 2013).

On the other hand, after growth in xylan medium, we observed that the shift in taxonomic composition (**Figure 2C**) was not followed by a significant change of the functional potential of the microbial assemblages (**Figure 2B**). This can be explained by different substrate preferences of species with similar functional potential, such as *Sphingobacterium* and *Cellulomonas*. *Sphingobacterium* increased after growth in xylan medium and it has been reported in various plant biomass-degrading microbial consortia and was found to secrete endo-beta-1,4-xylanases during growth on wheat straw (Jiménez et al., 2015). *Cellulomonas* strains have also been found to secrete xylanases (Sánchez-Herrera et al., 2007; Lisov et al., 2017). However, in the growth conditions used in our study, *Cellulomonas* seems to be outcompeted by *Sphingobacterium* when xylan is the sole carbon source. The original consortia were enriched in genes encoding exoglucanases, endoglucanases, and beta-glucosidases. Three of the original consortia were enriched in filter paper, which is composed of crystalline cellulose, and five consortia were bred in poplar wood chips, which is mainly composed of cellulose. Therefore it suggests that the observed association of *Cellulomonas* with the original consortia is related to their preference to degrade cellulose (Meinke et al., 1993).

Resource partitioning among distinct bacterial phyla has been demonstrated in soil microbial communities and is a major driver of microbial diversity (Wilson and Lindow, 1994; Goldfarb et al., 2011; Kramer et al., 2016). Our findings provides strong evidence that distinct players in lignocellulolytic microbial consortia have distinct substrate preferences. These bacteria are promising candidates for future studies of engineered microbial consortia in lignocellulose degradation. Studies have observed an increase in degradation rates in mixed cultures, when compared to monocultures (Tiunov and Scheu, 2005; Evans et al., 2017). Identifying key degraders of each plant cell wall polymer is the first step toward the development of engineered microbial consortia for an “optimal” plant biomass degradation. Using metagenomics, we confirmed the major role of *Pseudomonas* during degradation of lignin-derived aromatic compounds. Additionally, *Sphingobacterium* is identified as a good candidate for xylan degradation and *Cellulomonas* seems to play a crucial role during aerobic degradation of cellulose in complexes substrates.

## Author Contributions

CC designed and performed the experiments and analyses and also wrote the manuscript. HF performed the bioinformatics analysis and wrote the manuscript. CC designed and wrote the manuscript.

## Conflict of Interest Statement

The authors declare that the research was conducted in the absence of any commercial or financial relationships that could be construed as a potential conflict of interest.

## Footnotes

1 http://www.jgi.doe.gov

2 http://folk.uio.no/ohammer/past/

3 http://kiwi.cs.dal.ca/Software/STAMP
