# Supplementary figures and images for: Substrate Shift Reveals Roles for Members of Bacterial Consortia in Degradation of Plant Cell Wall Polymers
Source: Front Microbiol. 2018 Mar 1;9:364. doi: 10.3389/fmicb.2018.00364 (PMC5839234; doi:10.3389/fmicb.2018.00364)

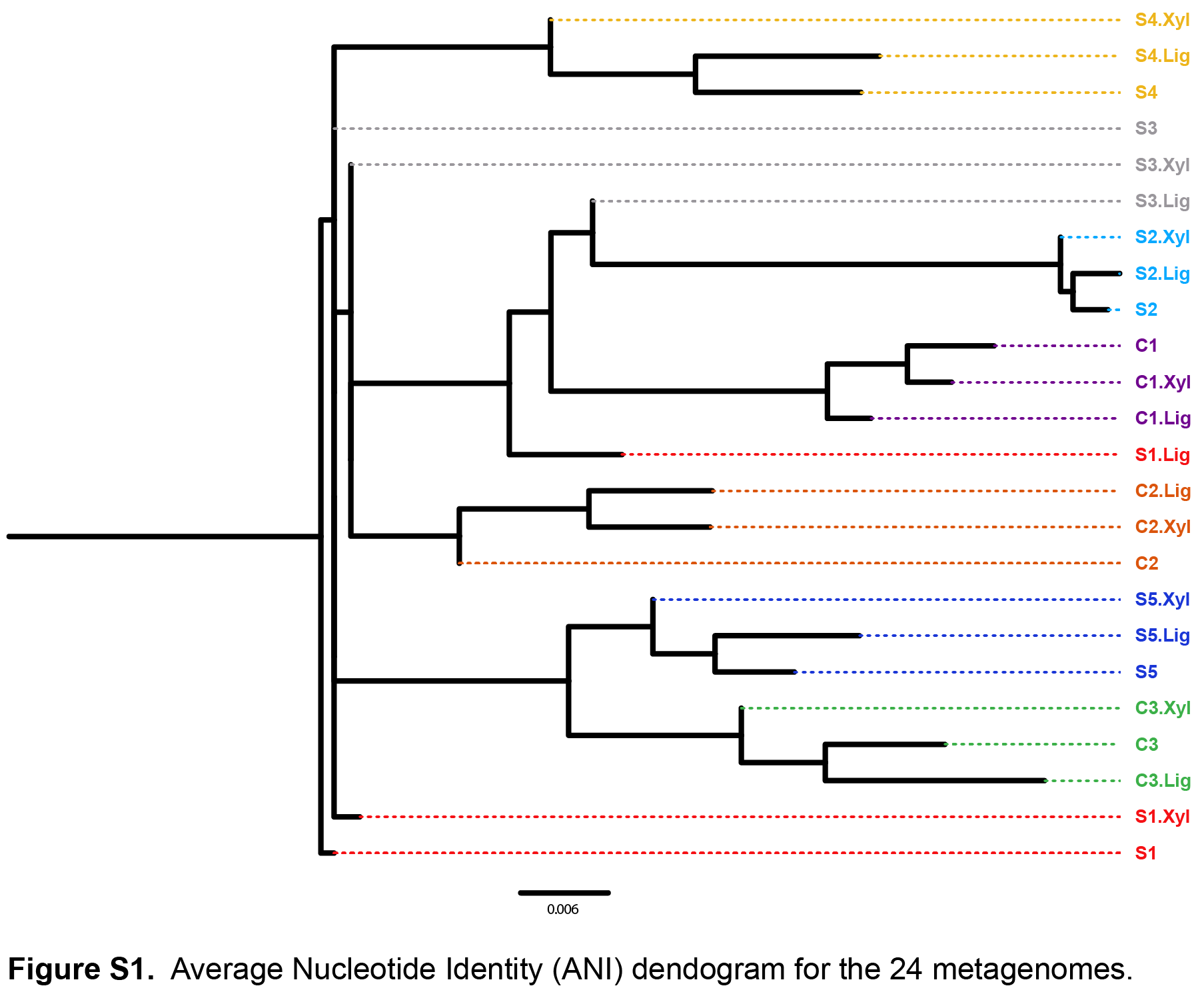

Supplement: Supplementary file 4 [file Image_1.tif]
